# Prevalence of post-stroke poor sleep quality: a meta-analysis of Pittsburgh Sleep Quality Index results

**DOI:** 10.3389/fneur.2025.1676047

**Published:** 2026-01-19

**Authors:** Yu Zhou, Bi Guan, Rong Tang, Qiongyao Zhong, Liangnan Zeng

**Affiliations:** ^1^Department of Operating Room, Chengdu Fifth People’s Hospital, Chengdu, China; ^2^Department of Nursing, Chengdu Fifth People’s Hospital, Chengdu, China; ^3^Department of Orthopedics, Chengdu Fifth People’s Hospital, Chengdu, China; ^4^Department of Neurology, Chengdu Fifth People’s Hospital, Chengdu, China; ^5^Department of Nursing, Hospital of Chengdu University of Traditional Chinese Medicine, Chengdu, China

**Keywords:** cerebrovascular accident, meta-analysis, poor sleep quality, prevalence rate, PSQI

## Abstract

**Objective:**

To assess the prevalence of post-stroke poor sleep quality using the Pittsburgh Sleep Quality Index (PSQI).

**Methods:**

This study was conducted according to the Preferred Reporting Items for Systematic Reviews and Meta-Analyses (PRISMA) guidelines. Following the PICO framework, a systematic search was conducted in PubMed, CINAHL, Cochrane Library, and Web of Science for relevant cohort, case-control, and cross-sectional studies published up to October 2024. The retrieved literature was then meta-analyzed using Stata 13.0 software.

**Results:**

Eighteen studies were reviewed, showing a total poor sleep quality prevalence of 55% (95%CI = 0.47 to 0.62) and a PSQI score is 8.12 (95%CI = 6.71 to 9.53). Compared to normal people, stroke patients sleep onset latency (SL) was prolonged by 1.36 min (95%CI = 0.82 to 1.90), sleep efficiency (SE) decreased by 1.48% (95%CI = −0.20 to −0.92), and periodic leg movements per hour of sleep (PLMI) increased by 1.07 per hour (95%CI = 0.56 to 1.59). Subgroup analysis showed that, compared with hemorrhagic stroke patients, ischemic stroke patients had higher incidence of poor sleep quality at 52% (95%CI = 0.24 to 0.86); the incidence of poor sleep quality was 59% (95%CI = 0.49 to 0.70) higher in chronic stroke patients compared to acute and subacute stroke patients; the incidence of poor sleep quality was 61% (95%CI = 0.51 to 0.71) higher in community stroke patients than in hospitalized stroke patients; and the incidence of poor sleep quality was 59% (95%CI = 0.58 to 0.61) higher in stroke patients in developing countries than those in developed countries.

**Conclusion:**

Current evidence suggests that quality of sleep worsens after a stroke, with symptoms being widespread. Factors such as stroke type, stroke phase, clinical setting, and research region can all impact sleep quality after stroke. These findings underscore the importance of monitoring sleep quality in these populations and implementing appropriate preventive and interventional strategies.

**Systematic review registration:**

https://www.crd.york.ac.uk/PROSPERO, identifier CRD420251161167.

## 1 Introduction

Stroke remains the primary cause of long-term functional impairment, exerting profound physical and psychological effects on survivors and their families. It ranks among the top three causes of death and premature disability worldwide (1–3). While stroke incidence has declined in developed countries, it has risen in low- and middle-income countries over the past two decades (4). Survivors often face chronic sequelae, including mobility, cognition, language, and communication impairments; emotional disturbances; poor sleep quality; difficulties in daily activities; and social isolation (5).

Up to 60% of stroke patients experience concomitant insomnia (6). These disorders significantly impact stroke rehabilitation and can worsen pre-existing conditions like hypertension and diabetes, thereby reducing patients’ quality of life (7–10). The pathophysiological mechanisms behind post-stroke poor sleep quality are not fully understood, but potential factors include: ① Due to damage or dysfunction of specific brain regions, leading to abnormalities in sleep–wake regulation, orexin/arousal pathways, and 5-hydroxytryptamine (5-HT) levels, insomnia occurs directly (11, 12); ② Abnormal upper airway movements cause sleep-disordered breathing, with upper airway muscle relaxation reaching its highest degree during rapid eye movement sleep, causing drooping of the jaw or hypopharyngeal region, increasing upper airway resistance, and reducing alveolar ventilation, resulting in sleep apnea (13); and ③ Post-stroke patients experience psychological factors such as excessive focus on their own condition and feelings of loss from leaving their work, which further aggravate their mental burden and exacerbate sleep problems (14).

Studies examining the relationship between poor sleep quality and cerebrovascular accidents have revealed a high prevalence of post-stroke poor sleep quality, with significant discrepancies in reported results due to varying measurement criteria (15). Currently, assessment tools such as the PSQI (16), Insomnia Severity Index (ISI) (17), Epworth Sleepiness Scale (ESS) (18), Athens Insomnia Scale (AIS) (19), and polysomnography (PSG) (20) are used to evaluate quality of sleep in patients, each with its own strengths and limitations. The PSQI is a frequently used sleep quality questionnaire that has been shown to be reliable and valid in many populations The PSQI is a subjective measure of sleep which assesses nighttime sleep quality in the past month, was used. It consists of 19 items, with the total score ranging from 0 to 21, where a score of >5 indicates poor sleep quality. This cut-off has been shown to have high specificity and sensitivity for distinguishing insomnia patients and controls (21). Many studies define a PSQI score >5 as poor sleep quality. Based on this, our study performs a meta-analysis of research using the PSQI to evaluate quality of sleep, aiming to investigate the prevalence of post-stroke poor sleep quality. The findings are expected to inform targeted prevention and management strategies for patients experiencing poor sleep quality post-stroke.

## 2 Methods

### 2.1 Literature search

**Figure 1:**
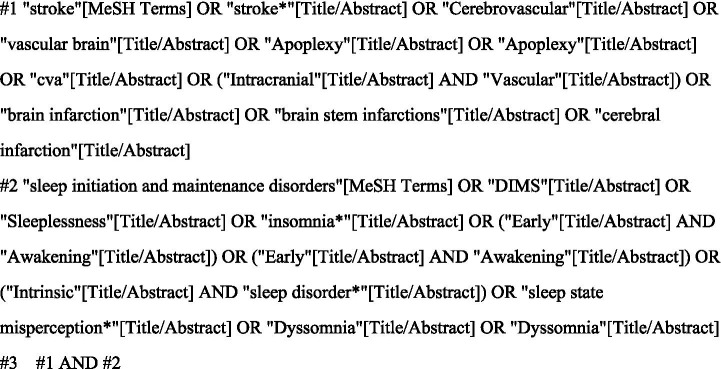
PubMed search strategy.

This study was conducted according to the PRISMA guidelines. The search strategy followed the PICOS framework. Two researchers, systematically trained in evidence-based practice, independently conducted literature searches on PubMed, CINAHL, Cochrane, Web of Science, Embase, and PsycINFO. Using PubMed as a representative example (see Figure 1), the researchers utilized a combination of free-text terms and controlled vocabulary to ensure comprehensive retrieval. The search covered the entire database history up to October 1, 2024, supplemented by manual tracking of references from the retrieved articles.

### 2.2 Study selection

Inclusion criteria: (1) Study designs included cohort studies, cross-sectional studies, and case-control studies. (2) The study subjects were stroke patients who met the diagnostic criteria outlined in the Diagnostic Criteria for Cerebrovascular Diseases. (3) The studies which were considered utilized the Pittsburgh Sleep Quality Index to assess sleep quality. (4) The primary outcome measure was the prevalence rate of post-stroke poor sleep quality. (5) The literature review concentrated on peer-reviewed and published English-language articles.

Exclusion criteria: (1) Conference abstracts; (2) literature lacking complete data reports or full-text availability; (3) duplicate publications; and (4) studies involving transient ischemic attack or subarachnoid hemorrhage were excluded. Studies with a “mixed” population (e.g., stroke and TIA), where results had not been reported separately for stroke, were included if the overall stroke sample exceeded 80%.

### 2.3 Data extraction

Literature screening and data extraction were independently performed by two researchers with evidence-based training. Discrepancies were resolved through discussion or consultation with a third researcher. EndNote software was utilized for deduplication. Preliminary screening involved reviewing titles and abstracts to exclude clearly irrelevant literature, followed by a full-text review to determine final inclusion. Original authors were contacted when necessary. Extracted data included: literature characteristics (author, publication year, and country); demographic features (age, sex/gender, BMI, sample size, smoking history, and alcohol consumption history); follow-up duration; stroke characteristics (stroke type, lesion location, lesion count, stroke assessment tool, and stroke score); comorbidities (anxiety assessment tool/score, depression assessment tool/score, diabetes mellitus, heart disease, and hypertension); and sleep parameters (prevalence rate of poor sleep quality, PSQI score, and objective data from Multi-Dimensional Sleep Mapping).

### 2.4 Quality assessment

A quality assessment of the literature was independently conducted by two researchers trained in evidence-based practice, and discrepancies were resolved through discussion or adjudication by a third researcher. Appropriate quality assessment methods were selected based on study designs. For cross-sectional studies, the risk of bias was evaluated using the assessment criteria recommended by the Agency for Health Care Research and Quality (AHRQ) (22). This 11-point scale assigns 1 point for “Yes” responses and 0 points for “No” or “Unclear” responses. Studies scoring 8–11 points were classified as high quality, 4–7 points as moderate quality, and 0–3 points as low quality. Studies scoring below 4 points are considered low-quality literature. Case–control studies and cohort studies were assessed using the Newcastle-Ottawa Scale (NOS) (23), which primarily consists of three categories and eight assessment items. The evaluation covers three aspects: sample selection, comparability between groups, and exposure factors. Each item scores 1 point, except for the comparability between groups criterion, which carries 2 points. The total NOS score ranges from 0 to 9 points: studies scoring 0–4 points were considered low-quality literature, 5–6 points as medium-quality, and 7–9 points as high-quality literature, with higher scores indicating better methodological quality. The studies included in this research were all cross-sectional studies.

### 2.5 Statistical analysis

Statistical analysis was performed using Stata 13.0 software. Measurement data were expressed as standardized mean difference (SMD) and weighted mean difference (WMD). The proportion of poor sleep quality measured in all included studies was extracted. Before pooling prevalence estimates, the variance of the raw prevalence from each included study was stabilized by using the Freeman-Tukey double arc-sine transformation (24). When the number of studies included was less than five, a fixed-effects model was used. When the number of studies included was greater than or equal to five, heterogeneity among study outcomes was first assessed (25). In cases where *p* > 0.1 and *I*^2^ < 50%, a fixed-effects model was applied; however, when *p* ≤ 0.1 or *I*^2^ ≥ 50%, indicating high heterogeneity, a random-effects model was used.

Subgroup analyses were used to explore the source of potential heterogeneity and were calculated based on categorical variables (e.g., cut-off values, country, study region, clinical environment, stroke period, and stroke type). Moreover, these subgroup analyses were conducted when there were at least two studies in each subgroup.

Sensitivity analysis was performed using the one-by-one exclusion method for individual studies. If sources of heterogeneity could not be identified, descriptive analysis was conducted. Funnel plots and Egger’s test were used to assess publication bias, with *p* < 0.05 considered statistically significant.

## 3 Results

### 3.1 Literature search results

**Figure 2:**
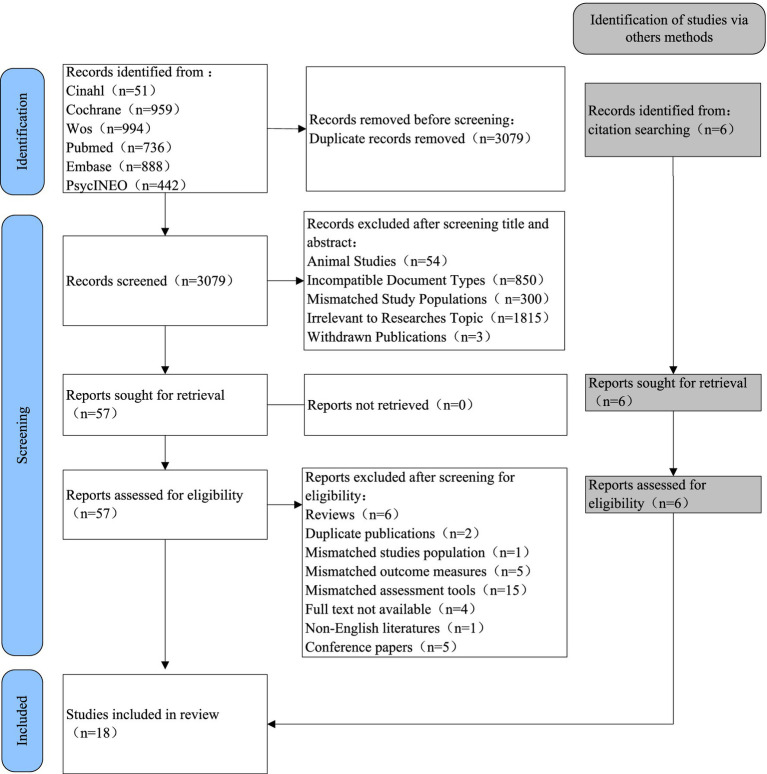
Literature screening flowchart.

The initial search identified 4,074 relevant articles. After removing duplicates, 3,079 articles remained. Following title and abstract screening, 61 articles underwent full-text review (reasons for exclusion included irrelevance to research topic, mismatched study population, and mismatched assessment tools), and 19 were ultimately included in the data analysis. The study selection process is shown in Figure 2.

### 3.2 Basic characteristics and methodological quality of included studies

Table 1 summarizes the characteristics of the included studies. Of the 19 cross-sectional studies, AHRQ scores ranged from 7 to 11 points, with 17 classified as high quality (26–41) and two as moderate quality (42, 43). Detailed quality assessments are provided in Table 1.

**Table 1:** Basic characteristics of included studies.

Author	Country	Sample	Poor sleep quality	PSQI	PSQI cut-off	Stroke period	Clinical environment	Stroke type	Male/female	Years	BMI	Comorbid hypertension	Comorbid diabetes mellitus	Comorbid heart disease	History of alcohol consumption	Smoking history	Quality score
Ho LYW (26) (2021)	China	112	72	7.51 ± 3.85	>5	Chronic phase	Community environment	–	38/74	64.18 ± 5.77	–	–	–	–	–	–	10
Zhang S (27) (2014)	China	223	105	–	>7	–	Hospital environment	Infarction	170/53	38.26 ± 6.351	–	104	44	–	111	97	9
Xie H (28) (2023)	China	205	74	–	>5	Chronic phase	Community environment	Infarction	138/67	65.36 ± 11.98	–	144	73	–	77	78	9
Sonmez I (29) (2019)	Cyprus	50	32	9.92 ± 5.55	>5	–	Hospital environment	Infarction, hemorrhage	28/22	69.92 ± 9.03	–	–	–	–	–	41	9
He W (30) (2022)	China	290	90	–	>5	Subacute phase	Hospital environment	Infarction	145/145	63.24 ± 9.89	24.26 ± 3.13	186	70	19	85	108	8
Rangel MFA (31) (2024)	Brazil	65	44	8.4 ± 4.3	>5	Chronic phase	Community environment	Infarction, hemorrhage	35/30	59.8 ± 12.9	28.2 ± 5.0	49	23	–	–	10	10
Kim KT (32) (2017)	South Korea	241	79	–	>5	Acute phase	Hospital environment	Infarction	146/95	64.17 ± 11.89	24.38 ± 3.62	147	73	–	–	117	8
Tayade K (34) (2023)	India	103	74	8 ± 4.51	>5	Subacute phase, chronic phase	Hospital environment	Infarction, hemorrhage	76/27	50.7 ± 11.7	26.2 ± 4.2	57	26	6	36	35	8
Kim J (33) (2015)	South Korea	80	57	8.1 ± 4.1	>5	Acute phase	Hospital environment	Infarction, hemorrhage	54/26	63.8 ± 13.6		36	17				9
Niu S (35) (2023)	China	530	311	–	>8	Subacute phase	Hospital environment	Infarction	377/135	63.24 ± 10.29	24.33 ± 2.97	325	241	64	217	204	10
Mansour AH (36) (2020)	Egypt	75	46	–	>5	–	Hospital environment	–	34/41	59.3 ± 5.34	–	–	–	–	–	–	9
Karaca B (37) (2016)	Türkiye	23	9	5.2 ± 4.25	>5	–	Hospital environment	–	14/9	60.2 ± 9.9	27.2 ± 3.1	17	7	3	–	–	9
Iddagoda MT (38) (2020)	Australia	104	31	–	>5	–	Hospital environment	Infarction, hemorrhage	55/49	80.11 ± 6.82	–	–	–	–	–	–	9
Fan XW (39) (2022)	China	1,619	1,137	–	>5	Subacute phase, chronic phase	Hospital environment, community environment	–	1173/446	60.8 ± 10.7	–	1,020	375		274	580	9
Zhang Y (40) (2022)	China	1,210	719	–	>5	Acute phase, subacute phase, chronic phase	Hospital environment	Infarction, hemorrhage	837/373	60.45 ± 10.33	–	882	366	243	536	612	11
Wu W (41) (2016)	China	27	27	6.6 ± 0.6	>5	Subacute phase, chronic phase	Hospital environment, community environment	–	19/8	61.4 ± 2.5	25.0 ± 0.6	17	11	–	11	12	8
Sterr A (42) (2018)	United Kingdom	19	14	6.9 ± 3.1	>5	–	Community environment	–	13/6	63.5 ± 8.0	26.9 ± 3.6	–	–	–	–	–	7
Wang H (43) (2022)	China	23	23	14.17 ± 3.63	>5	–	Hospital environment	Infarction	12/11	62.48 ± 8.74	–	–	–	–	–	–	7

### 3.3 Meta-analysis results

#### 3.3.1 Evaluating post-stroke quality of sleep symptoms using PSG

Two studies using PSG analyzed sleep quality. Through fixed-effects model meta-analysis, compared to the control group, post-stroke patients showed: sleep onset latency (SL) prolonged by 1.36 min (95%CI = 0.82 to 1.90), sleep efficiency (SE) decreased by 1.48% (95%CI = −0.20 to −0.92), periodic leg movements per hour of sleep (PLMI) increased by 1.07 per hour (95%CI = 0.56 to 1.59), and wake after sleep onset (WASO) with no statistically significant difference at 0.44 min (95%CI = 0.03 to 0.90). Specific details are shown in Table 2.

**Table 2:** Results of sleep symptoms after stroke.

Variables	Number of included studies	Heterogeneity test		WMD	95%CI
		*I*^2^ value (%)	*p*-value		
SL	2	95.2%	*p* < 0.001	1.36	0.82 to 1.90
SE	2	96.1%	*p* < 0.001	−1.48	−0.20 to −0.92
PLMI	2	37.0%	*p* < 0.001	1.07	0.56 to 1.59
WASO	2	94.2%	*p* = 0.208	0.44	0.03 to 0.90

#### 3.3.2 Assessing the prevalence of poor sleep quality after stroke using the PSQI

**Figure 3:**
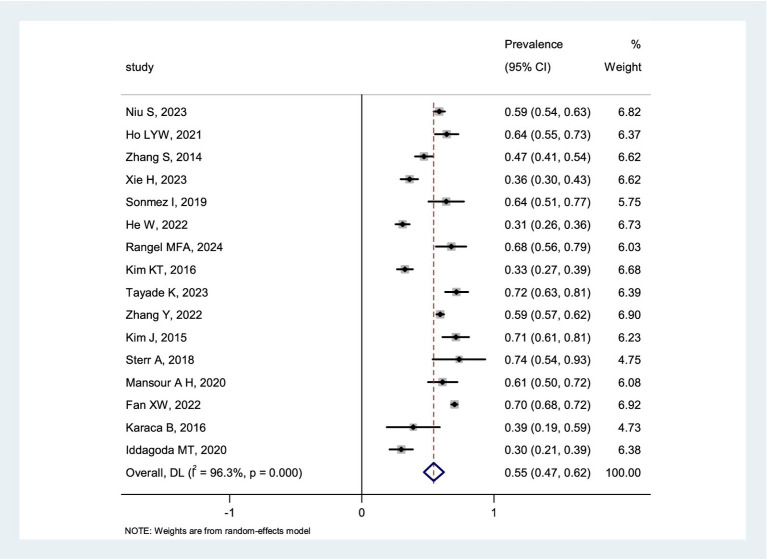
Forest plot of the prevalence rate of post-stroke poor sleep quality.

Sixteen studies reported on the prevalence of post-stroke poor sleep quality. Considerable heterogeneity was observed among these studies (*I*^2^ = 96.3%, *p* < 0.1). Using a random-effects model for pooling, the meta-analysis found an overall prevalence rate of 55% (95%CI = 0.47 to 0.62). A forest plot illustrating the prevalence rates of post-stroke poor sleep quality is shown in Figure 3.

#### 3.3.3 Assessing post-stroke quality of sleep using the PSQI scale

**Figure 4:**
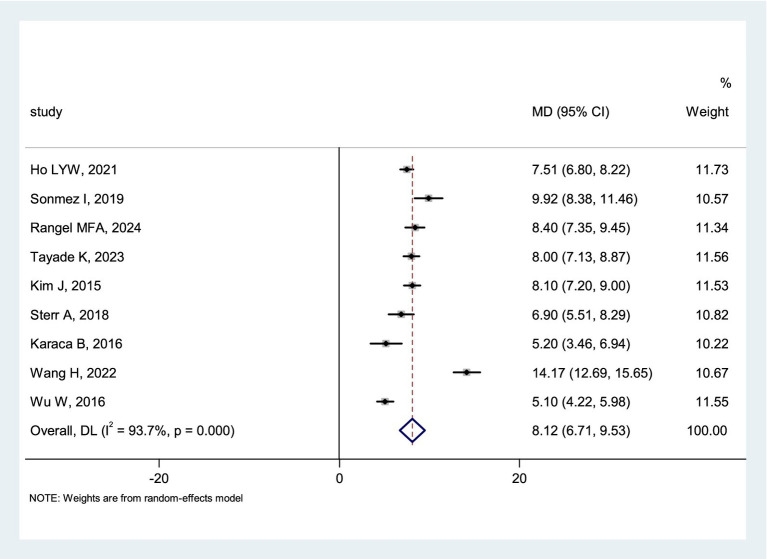
Forest plot of post-stroke PSQI score.

Nine studies reported post-stroke PSQI scores. Significant heterogeneity was observed among these studies (*I*^2^ = 93.7%, *p* < 0.1). A random-effects model was used for pooling. The meta-analysis showed a mean post-stroke PSQI score of 8.12 points (95%CI = 6.71 to 9.53). The forest plot for post-stroke PSQI score is presented in Figure 4.

#### 3.3.4 The impact of stroke type on poor sleep quality prevalence

Studies have compared the impact of stroke types on the incidence of poor sleep quality. Subgroup analyses were conducted based on cerebrovascular accident type. Significant heterogeneity persisted across all subgroups (*I*^2^ > 50%, *p* < 0.1), and a random-effects model was applied for pooling. The prevalence was 52% for ischemic stroke and 50% for hemorrhagic stroke. The incidence of poor sleep quality in cases of atherosclerotic cerebral infarction of the artery was 63%. For cardioembolic infarction, poor sleep quality incidence was 65%, while for other cases of acute ischemic stroke, it was 68%. Only one study evaluated the impact of lacunar and non-lacunar ischemic stroke on poor sleep quality incidence, finding that lacunar ischemic stroke patients had a poor sleep quality incidence of 21.43%, while non-lacunar ischemic stroke patients showed an incidence of 11.05%. Details are provided in Table 3.

**Table 3:** Meta-analysis of stroke types on the prevalence rates of post-stroke poor sleep quality.

Stroke type	Number of included studies	Heterogeneity test		Sample size	Prevalence (95%CI)
		*I*^2^ value (%)	*p*-value		
Ischemic cerebral stroke	3	96.0%	<0.001	213	0.52 (0.24–0.86)
Cerebral hemorrhagic stroke	3	0.0%	0.625	44	0.50 (0.35–0.65)
Large artery atherosclerosis	2	98.6%	<0.001	445	0.63 (0.59–0.68)
Cardioembolism	2	79.5%	<0.001	119	0.65 (0.56–0.73)
Small vessel occlusion/others	2	96.0%	<0.001	1,260	0.68 (0.65–0.71)

#### 3.3.5 Impact of comorbidities on the prevalence of poor sleep quality after stroke

Subgroup analyses were conducted based on comorbidity. Significant heterogeneity persisted across all subgroups (*I*^2^ > 50%, *p* < 0.1), and a random-effects model was applied for pooling. When combined with hypertension, the prevalence was 52%; with diabetes mellitus, 55%; and with heart disease, 57%. Details are provided in Table 4.

**Table 4:** Meta-analysis of comorbidities on poor sleep quality prevalence after stroke.

Subgroup	Number of included studies	Heterogeneity test		Sample size	Prevalence (95%CI)
		*I*^2^ value (%)	*p*-value		
Comorbid hypertension					
Yes	4	94.9%	<0.001	802	0.52 (0.48–0.55)
No	4	95.8%	<0.001	365	0.41 (0.15–0.58)
Comorbid diabetes mellitus					
Yes	4	89.6%	<0.001	661	0.55 (0.51–0.60)
No	4	95.9%	<0.001	1,036	0.45 (0.41–0.48)
Comorbid heart disease					
Yes	2	0.0%	<0.001	77	0.57 (0.46–0.67)
No	2	98.4%	<0.001	782	0.47 (0.44–0.50)

#### 3.3.6 Impact of demographic characteristics on the prevalence of poor sleep quality after stroke

Significant heterogeneity persisted across all subgroups (*I*^2^ > 50%, *p* < 0.1), and a random-effects model was applied for pooling. The results indicated that the prevalence of post-stroke poor sleep quality was 59% in the chronic phase, 55% in the acute phase, and 45% in the subacute phase. Moreover, prevalence was found to be 61% in community environments compared with 53% in hospital environments, while being 59% in developing countries compared with 44% in developed countries (Table 5).

**Table 5:** Meta-analysis of demographic characteristics on post-stroke poor sleep quality prevalence.

Subgroup	Number of included studies	Heterogeneity test		Sample size	Prevalence (95%CI)
		*I*^2^ value (%)	*p*-value		
Sex/gender					
Male	6	94.2%	<0.001	674	0.48 (0.33–0.62)
Female	6	95.1%	<0.001	647	0.51 (0.30–0.71)
Age					
≥60 years	4	87.2%	<0.001	466	0.62 (0.49–0.74)
<60 years	12	97.1%	<0.001	4,522	0.52 (0.43–0.61)
BMI					
<25	3	97.6%	<0.001	210	0.44 (0.42–0.47)
≥25	4	67.1%	=0.028	1,100	0.66 (0.61–0.74)
Alcohol history					
Yes	4	94.0%	<0.001	468	0.55 (0.50–0.59)
No	4	94.5%	<0.001	699	0.45 (0.42–0.49)
Smoking history					
Yes	7	91.8%	<0.001	424	0.55 (0.39–0.71)
No	7	93.8%	<0.001	848	0.47 (0.31–0.63)
PSQI cut-off					
>5	14	96.7%	<0.001	4,528	0.55 (0.46–0.64)
>7	2	88.3%	=0.003	753	0.53 (0.42–0.66)
Stroke period					
Acute phase	3	97.6%	<0.001	1,863	0.55 (0.34–0.75)
Subacute phase	2	98.4%	<0.001	820	0.45 (0.18–0.72)
Chronic phase	6	96.4%	<0.001	3,211	0.59 (0.49–0.70)
Clinical environment					
Hospital environment	13	98.9%	<0.001	6,232	0.53 (0.41–0.65)
Community environment	6	95.5%	<0.001	3,230	0.61 (0.51–0.71)
Study region					
Developing countries	13	96.7%	<0.001	4,776	0.59 (0.58–0.61)
Developed countries	3	92.7%	<0.001	173	0.44 (0.37–0.51)

### 3.4 Sensitivity analysis and publication bias

**Figure 5:**
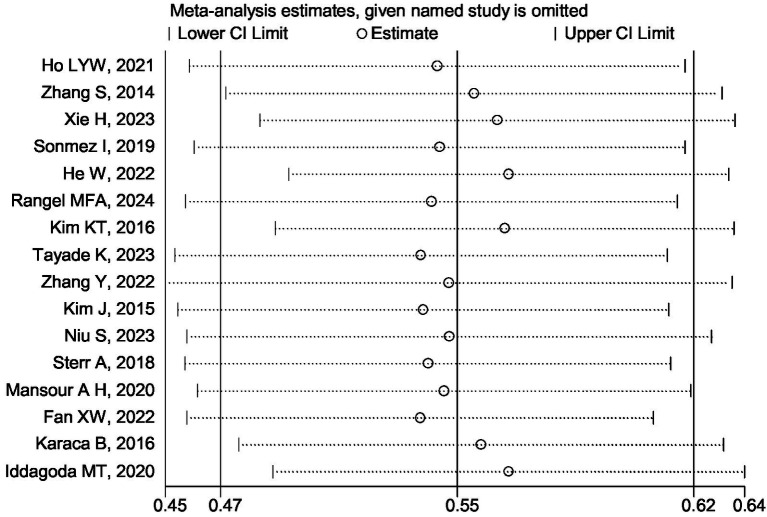
Sensitivity analysis of post-stroke quality of sleep.

**Figure 6:**
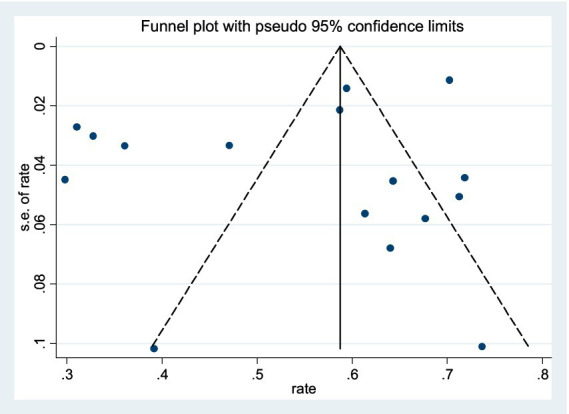
Funnel plot for post-stroke quality of sleep.

Sixteen analyses were conducted by sequentially removing individual studies from the pooled data. The overall results remained largely consistent, demonstrating the robustness of the meta-analysis findings, as illustrated in Figure 5. Publication bias was assessed using a funnel plot analysis of poor sleep quality prevalence rates. Although the funnel plot displayed an asymmetrical distribution of scatter points (Figure 6), Egger’s test revealed no statistically significant difference (*p* = 0.186), indicating a low risk of publication bias.

## 4 Discussion

This meta-analysis of two studies using PSG to assess post-stroke poor sleep quality symptoms found that post-stroke patients experienced prolonged sleep onset latency (patients: range 23.1–23.9, mean 23.43; vs. controls: range 9.2–15.9, mean 13.46); reduced sleep efficiency (patients: range 72–78.8, mean 76.0; vs. controls: range 79.1–88.7, mean 82.62); increased periodic leg movements per hour (patients: range 12.08–13.7, mean 13.04; vs. controls: range 2.2–8.07, mean 5.92); and a trend toward increased WASO 9 (patients: range 21.4–102.7, mean 54.89; vs. controls: range 21.1–72.1, mean 53.54). The data suggest that stroke patients have poor sleep continuity and reveal subtle changes in sleep structure, though these observations are not yet sufficient to draw definitive conclusions. These changes may result from alterations in awakening and cortical-thalamic-cortical oscillations, with slow oscillations from intact cortical networks serving as envelopes for other brain oscillations like slow-wave activity or sleep spindles. Cortical lesions might disrupt this process, primarily affecting sleep continuity (44). Furthermore, the psychological impact of stroke could potentially increase cognitive (psychosocial) arousal, thereby leading to even poorer sleep continuity. Future research is anticipated to combine PSG with structural neuroimaging studies.

This study conducted a meta-analysis of 16 studies which reported on the prevalence of post-stroke poor sleep quality, revealing a pooled prevalence rate of 55%, clearly higher than the conclusions drawn from a Hinz A survey of the general population (45). Additionally, this study performed a meta-analysis of nine studies reporting post-stroke PSQI scores, finding that the average PSQI score in post-stroke patients was 8.12 points. These findings highlight the prominence of post-stroke poor sleep quality, which significantly impacts the quality of life in patients with cerebrovascular accidents. This may be due to factors such as neural functional deficits, overall body functional status, negative emotions, environmental factors, or treatment factors which have affected the patient’s sleep quality. Compared with other complications, the subtle nature of poor sleep quality symptoms makes them more likely to be overlooked by healthcare professionals in clinical practice. However, post-stroke poor sleep quality has a significantly higher prevalence rate than other stroke complications such as delirium, aspiration pneumonia, and venous thromboembolism (46, 47). Therefore, it is recommended that future clinical practice place greater emphasis on poor sleep quality and promote screening initiatives to facilitate early intervention, thereby mitigating or improving patients’ sleep disturbances and reducing adverse outcomes.

The prevalence of post-stroke poor sleep quality is generally lower in ischemic stroke patients compared to those with cerebral hemorrhagic stroke. However, this study found a higher prevalence of poor sleep quality for individuals experiencing ischemic stroke (52%) than those with hemorrhagic stroke (50%). Considering the limited number of studies conducted on stroke subtypes, this finding is tentative and needs to be replicated in future research.

In addition, this study found that the prevalence of poor sleep quality after stroke in developing countries (59%) was higher than in developed countries (44%). This may be related to insufficient stroke healthcare resources and fewer trained healthcare professionals in developing countries compared to developed countries, all of which can lead to adverse health outcomes, including poor sleep quality (48). It was found in this study that the prevalence of poor sleep quality in the chronic phase after stroke was 59%, higher than in the acute phase (55%) and subacute phase (45%). This is inconsistent with previous research, which found the prevalence of poor sleep quality during the acute, subacute, and chronic phases to be 40.7, 42.6, and 35.9% (49), respectively. This discrepancy may be due to differences in the types of sleep problems investigated across different studies. The present study found a significant difference in the prevalence of poor sleep quality in different clinical settings, with a higher prevalence of sleep disorders in community patients (61%) than in hospitalized patients (53%). Stroke treatment and rehabilitation are significantly associated with the sleep quality of stroke survivors (50). Hospitalized patients have more timely and easier access to medical resources compared to community patients; thus, the incidence of sleep-related problems is relatively lower.

The prevalence of post-stroke poor sleep quality was higher in patients aged ≥60 years (62%) compared to those aged <60 years (52%) which may be related to sleep apnea. One study suggests that the prevalence of sleep apnea among people aged 30–70 increases with age (51). Sleep apnea may lead to intermittent hypoxia, increased sleep awakenings (52), shortened sleep time, and decreased sleep quality (53). Future research exploring the association between sleep apnea and stroke is anticipated. Patients with BMI ≥ 25 showed a higher prevalence of post-stroke poor sleep quality (68%) compared to those with BMI < 25 (31%). In overweight/obese individuals, increased tongue fat deposition narrows the pharyngeal airway, and abdominal obesity can alter sleep structure, leading to sleep deprivation. This further triggers increased growth hormone-releasing peptide secretion and decreased leptin, promoting increased appetite. Increased appetite and weight gain further exacerbate poor sleep quality, creating a vicious cycle (54). Therefore, in stroke patients with obesity, it is necessary to control patient healthy diet and physical activities.

### 4.1 Strengths and limitations of the study

This study adopted a comprehensive review protocol and followed the PRISMA guidelines. Multiple electronic databases were searched, and studies were screened, while data were extracted and evaluated using rigorous methods. Additionally, only research using the PSQI was included, to reduce research heterogeneity. However, this study still has the following limitations: (1) All of the literature included comprised cross-sectional studies. Due to differences in study design, research quality, sample sources, and sample sizes, substantial heterogeneity was observed among the studies. Prevalence is an average across highly heterogeneous studies and should not be interpreted as a precise estimate applicable to all settings. (2) The generalizability of the findings may be limited, as only English-language literature was included. (3) Since only some studies reported excluding patients with pre-existing poor sleep quality prior to enrollment, the potential influence of pre-existing poor sleep quality on our research outcomes could not be fully ruled out. (4) Due to the limited number of studies included, this research has not yet explored the relationships between post-stroke poor sleep quality prevalence rates, PSQI scores, and factors such as lesion location, lesion count, or stroke severity scores. (5) The PSQI is a self-reported sleep quality measurement method and may be less accurate than objective measurement methods.

## 5 Summary

This study found a high prevalence of poor sleep quality after stroke. The incidence of poor sleep quality is higher in developing countries, as well as higher for community-dwelling patients, patients in the chronic phase of stroke, those aged ≥60 years, and those with a BMI ≥ 25. Therefore, it is recommended that healthcare institutions at all levels pay attention to monitoring these populations and accelerate the implementation of comprehensive prevention and treatment strategies to reduce the prevalence of poor sleep quality in stroke patients and reduce adverse outcomes. Future research could further focus on the incidence and symptoms of poor sleep quality before stroke and investigate the impact of sleep apnea on new-onset stroke, recurrent stroke, and adverse stroke outcomes. Additionally, we look forward to future studies examining the differences in stroke subtypes from the perspective of quality of sleep, such as lacunar and non-lacunar ischemic strokes, atherosclerotic strokes, and cardiogenic embolic strokes, as well as exploring the relationship between post-stroke poor sleep quality and gastrointestinal symptoms and gut microbiota based on the brain-gut axis theory.

## Data Availability

The data analyzed in this study are not publicly available, but can be provided upon reasonable request. Requests to access these datasets should be directed to Yu Zhou, 2568963148@qq.com.
